# The Role of the Reactive Species Involved in the Photocatalytic Degradation of HDPE Microplastics Using C,N-TiO_2_ Powders

**DOI:** 10.3390/polym13070999

**Published:** 2021-03-24

**Authors:** Aranza Denisse Vital-Grappin, Maria Camila Ariza-Tarazona, Valeria Montserrat Luna-Hernández, Juan Francisco Villarreal-Chiu, Juan Manuel Hernández-López, Cristina Siligardi, Erika Iveth Cedillo-González

**Affiliations:** 1 Universidad Autónoma de Nuevo León, Facultad de Ciencias Químicas, Av. Universidad S/N Ciudad Universitaria, San Nicolás de los Garza C.P. 66455, Nuevo León, Mexico; aranza.vitalgr@uanl.edu.mx (A.D.V.-G.); valeria.lunahrn@uanl.edu.mx (V.M.L.-H.); juan.hernandezlz@uanl.edu.mx (J.M.H.-L.); 2 Department of Engineering “Enzo Ferrari”, University of Modena and Reggio Emilia, Via P. Vivarelli 10/1, 41125 Modena, Italy; mariacamila.arizatarazona@unimore.it (M.C.A.-T.); cristina.siligardi@unimore.it (C.S.); 3 Centro de Investigación en Biotecnología y Nanotecnología (CIByN), Facultad de Ciencias Químicas, Universidad Autónoma de Nuevo León, Parque de Investigación e Innovación Tecnológica, Km. 10 Autopista al Aeropuerto Internacional Mariano Escobedo, Apodaca 66629, Nuevo León, Mexico

**Keywords:** microplastics, HDPE, microbeads, photocatalysis, scavengers, C,N-TiO_2_, remediation, nanotechnology, plastic pollution, visible light photodegradation

## Abstract

Microplastics (MPs) are distributed in a wide range of aquatic and terrestrial ecosystems throughout the planet. They are known to adsorb hazardous substances and can transfer them across the trophic web. To eliminate MPs pollution in an environmentally friendly process, we propose using a photocatalytic process that can easily be implemented in wastewater treatment plants (WWTPs). As photocatalysis involves the formation of reactive species such as holes (*h*^+^), electrons (*e*^−^), hydroxyl (OH^●^), and superoxide ion (O_2_^●−^) radicals, it is imperative to determine the role of those species in the degradation process to design an effective photocatalytic system. However, for MPs, this information is limited in the literature. Therefore, we present such reactive species’ role in the degradation of high-density polyethylene (HDPE) MPs using C,N-TiO_2_. Tert-butanol, isopropyl alcohol (IPA), Tiron, and Cu(NO_3_)_2_ were confirmed as adequate OH^●^, *h*^+^, O_2_^●−^ and *e*^−^ scavengers. These results revealed for the first time that the formation of free OH^●^ through the pathways involving the photogenerated *e*^−^ plays an essential role in the MPs’ degradation. Furthermore, the degradation behaviors observed when *h*^+^ and O_2_^●−^ were removed from the reaction system suggest that these species can also perform the initiating step of degradation.

## 1. Introduction

Microplastics (MPs) are defined as plastic particles with a diameter ≤ 5 mm and are categorized as primary or secondary according to their origin [1]. Primary MPs are manufactured explicitly to that particle size to be used in cosmetic or personal hygienic products [1]. On the other hand, secondary MPs are derived from large plastic items’ fragmentation due to photolytic, mechanical, and biological degradation [1].

MPs pollution has increased so rapidly in recent years that it has become a global concern. Today, MPs can be found in a wide variety of sizes, shapes, and polymers in samples of soil, sand, air, water, wastewater, and snow all around the globe [1,2]. More importantly, due to their high specific surface area, MPs can adsorb persistent organic pollutants (POPs), hydrophobic organic contaminants (HOCs), pharmaceuticals, and heavy metals [1,2,3]. As MPs are frequently consumed by the biota present in aquatic and terrestrial ecosystems, these hazardous compounds can move easily through the trophic chain up to humans [1,2]. This has been evidenced by the presence of MPs in human stool [4] and even in the placenta [5]. Furthermore, the SARS-CoV-2 pandemic has increased MPs fibers’ presence in the environment due to the inadequate disposal of single-use plastic face masks [6].

In this context, there is an urgent need to develop remediation technologies to dissipate MPs pollution. The current methodologies for this include physical removal, biodegradation, and chemical treatments [7,8]. These latter include photocatalysis, a process that has been widely used to mineralize recalcitrant organic pollutants in aqueous and gaseous environments [9].

There are several significant advantages of using photocatalysis as a remediation technology to solve MPs pollution. Firstly, MPs can be entirely mineralized to CO_2_ and water. Only a small fraction is known to be converted into innocuous or less toxic chemical substances [10,11]. Secondly, photocatalysis can easily be adapted to wastewater treatment plants (WWTPs), identified as a potential source of MPs to aquatic and terrestrial ecosystems [1,2]. By coupling MPs photocatalysis to WWTPs, not only could the MPs level in discharge waters be significantly reduced, but the harmful problems that these pollutants cause to their facilities could also be avoided (inhibitory effects on activated sludge flocs, reduction of the diversity of biological communities and decline of the abundance of crucial microorganisms [12]). Finally, the photocatalytic process can be regarded as environmentally friendly since it can be performed under visible or solar light. Simultaneously, the photocatalyst can be prepared using renewable raw materials, respecting the sixth, seventh and ninth principles of Green Chemistry.

Photocatalysis has recently been proven to degrade low-density polyethylene (LDPE), high-density polyethylene (HDPE), polystyrene (PS), and polypropylene (PP) MPs in aqueous solutions [10,11,13,14,15,16,17,18,19]. However, complete degradation of any of these plastics has not been reported yet. To improve or optimize MPs degradation and be able to achieve their mineralization, different criteria must be considered, such as the photocatalyst’s properties, the pollutant’s characteristics and the reaction conditions, and the potential role of reactive species such as holes (*h*^+^), electrons (*e*^−^), and reactive oxygen species (ROS) that are formed along the process and could participate in the degradation reaction.

To date, the role of these reactive species in MPs degradation has been lightly investigated. For example, Nabi et al. [18] reported the "solid phase" photodegradation of 400 nm PS MPs using a Triton X-100 derived TiO_2_ nanoparticle film with ethylenediaminetetraacetic acid (EDTA), tert-butyl alcohol (TBA), and anaerobic conditions to check the role of holes, hydroxyl radicals (OH^●^), and oxygen (O_2_) in the degradation process. By monitoring the change in the PS particle’s diameter using scanning electron microscopy (SEM) analysis, their experiments demonstrated that the *h*^+^ was the dominant active specie in their reaction system when submitted under UV light for 6 h [18]. On the other hand, Jiang et al. [11] evaluated the photocatalytic degradation of a 1 g/L aqueous dispersion of HDPE MPs (200–250 μm) using a hydroxy-rich bismuth oxychloride photocatalyst and visible light (λ ≥ 420 nm). The role of *h*^+^, OH^●^, and superoxide ion radical (O_2_^●−^) in the degradation process was monitored by Fourier-transform infrared spectroscopy (FTIR) using methanol, isopropyl alcohol (IPA) and p-benzoquinone as scavengers. Results demonstrated that free OH^●^ formed through the reaction of adsorbed water with surface hydroxyl was the main reaction group involved in the degradation process [11].

These studies suggested that different reactive species can promote MPs’ degradation depending on the reaction conditions (solid- or aqueous-based reaction). However, there is still a need for a more in-depth analysis regarding the specific role that each one of those species may have on MPs degradation.

The present work is aimed to analyze the specific role of four reactive species in the photocatalytic degradation of primary HDPE MPs using C,N-TiO_2_ powders. To achieve this, tert-butanol, isopropyl alcohol, Tiron, and copper nitrate were used as OH^●^, *h*^+^, O_2_^●−^ and *e*^−^ scavengers, respectively. The reactions were performed in an aqueous solution because of its relevance for applying this technology in WWTPs. The C,N-TiO_2_ powders were chosen because they enable to perform the experiments in visible light and, get a better interaction between the dispersed photocatalyst and MPs [13]. It was found that all four species have an essential role in MPs degradation, suggesting for the first time that the photogenerated *e*^−^ plays an essential role in the photocatalytic degradation of HDPE MPs, mainly due to the formation of free OH^●^. Furthermore, it was demonstrated that the initiating step on MPs degradation is performed by OH^●^, *h*^+^ or O_2_^●−^ species.

## 2. Materials and Methods

### 2.1. MPs Obtainment and Characterization

Primary HDPE MPs were obtained from a commercial facial scrub using the extraction methodology proposed by Napper et al. [20]. HDPE MPs particle size and morphology were determined by optical microscopy (OM) using a light microscope (DME, Leica, Wetzlar, Germany) with a microscope camera (ICC50 W, Leica, Wetzlar, Germany). Morphology was also investigated by SEM coupled with energy dispersive X-ray spectroscopy (EDS), using a Thermo-Fisher Scientific FEI E-SEM (Quanta-200, FEI Company, Hillsboro, OR, USA). Before the analysis, the samples were coated with a gold layer of 10 nm. The type of polymer present in the sample was determined by attenuated total reflectance-Fourier-transform infrared spectroscopy (ATR-FTIR) in an infrared spectrometer (ALPHA II, Bruker, Ettlingen, Germany). The spectrum was collected by averaging 32 scans between 400 and 4000 cm^−1^ with a 4 cm^−1^ spectral resolution. The surface area was evaluated by nitrogen adsorption, using a surface area analyzer (Tristar II Plus 3.01, Micromeritics, Norcross, GA, USA) (bath temperature of 77.3 K, equilibration interval of 5 s, and degasification at 30 °C for 23 h).

### 2.2. C,N-TiO_2_ Preparation and Characterization

The C,N-TiO_2_ semiconductor was prepared by the synthesis procedure reported by Zeng et al. [21], with some modifications already described in previous work [13]. The crystalline composition was determined by X-ray diffraction (XRD), using a diffractometer with Cu Kα1 radiation, current of 40 mA, accelerating voltage of 40 kV, and 2θ-steps of 0.023° (D5000, SIEMENS, Munich, Germany). The chemical groups adsorbed in the semiconductor’s surface were identified by ATR-FTIR (ALPHA II, Bruker, Ettlingen, Germany), using the analysis conditions reported in Section 2.1. The presence of nitrogen and carbon in the TiO_2_ semiconductor was confirmed by elemental analysis using a CHNS/O analyzer with acombustion temperature of 975 °C and a reducing column with helium at 88 °C (2400 Series II, PerkinElmer, Waltham, MA, USA). The bandgap (*E*_g_) was calculated from the reflectance spectrum (Figure S1, Supplementary Materials) using the Kubelka-Munk equation (Equation S1). The spectrum was measured in an UV-vis/NIR spectrophotometer with BaSO_4_ (DEQ, Monterrey, Mexico) as reference material (V-670, JASCO, Tokyo, Japan). The microstructure was analyzed by field emission gun scanning electron microscopy (FEG-SEM)(Nova NanoSEM 450, FEI Company, Hilssboro, Oregon, USA). The BET surface area was evaluated by nitrogen adsorption, using a BET analyzer (Gemini 2380, Micromeritics, Norcross, GA, USA) (bath temperature of 77.3 K, equilibration interval of 10 s, and degasification at 110 °C).

### 2.3. Photocatalytic Experiments in the Absence and Presence of Scavengers

The photocatalytic experiments’ reaction conditions were carried out according to our previous work, which demonstrated the photocatalytic degradation of HDPE MPs using the C,N-TiO_2_ material [13]. Therefore, photocatalysis without scavengers was conducted in a Batch-type glass reactor, using 50 mL of a 0.4 wt./vol % HDPE MPs dispersion (pH 3) and a C,N-TiO_2_ load of 200 mg. The reactor was placed in a closed reaction chamber equipped with a constant temperature bath (set at 0 ± 2 °C) and irradiated for 50 h with a Slim LED IP65 50 W visible LED lamp (ARE-006, ARTlite, New Delhi, India) placed at 25 cm from the sample (57.2 ± 0.3 W/m^2^). MPs were dispersed in the experiments by continuous stirring at 350 rpm. After photocatalysis, the residual (degraded) MPs were separated from the reaction system and washed twice with distilled water to remove potential C,N-TiO_2_ traces that could remain attached. The photocatalytic experiments with the scavengers were performed according to this methodology, but adding reagent grade tert-butanol (0.2 mol, DEQ, Monterrey, Mexico), IPA (0.5 mol, DEQ, Monterrey, Mexico), 4,5-dihydroxy-1,3-benzenedisulfonic acid disodium salt “tiron” (2.18 × 10^−5^ mol, Sigma Aldrich, Ciudad de México, México) or copper nitrate (5.0 × 10^−3^ mol, DEQ, Monterrey, Mexico) as scavengers [22,23] (Table 1). The quantities of each scavenger were chosen based on previous reports [22,23] and, their concentrations were in excess respect to the HDPE MPs pollutants. Three replicates were performed for all the photocatalytic experiments, and the mean ± S.D. values are reported.

To determine the reactive species’ role in MPs photocatalysis, three analytical methods were used to monitor and confirm MPs degradation. These methods were particularly used as they are known to be effective independently of the presence of C,N-TiO_2_ traces. The first method used was gravimetry. This technique is commonly used due to its simplicity and represents a direct way to quantify the polymers’ degradation [24]. As expected, no presence of traces of C,N-TiO_2_ in the residual MPs was detected by this method. All mass measurements were performed in an analytical balance (CY224C, ACZET, Mumbai, India) after 15, 30, and 50 h of photocatalysis. Changes in MPs’ concentration were calculated and reported as wt./vol %. After 50 h of photocatalysis, MPs degradation was monitored by FTIR, which can detect polar functional groups, such as ketones and ester carbonyls (typical of oxidative degradation pathways [24]). Nevertheless, as higher intensity of the carbonyl band does not always represent more plastic degradation, this signal has to be compared to second reference band [25]. Consequently, the extent of oxidation during MPs degradation was quantified by calculating their carbonyl index (CI). CI quantifies the absorbance change for the carbonyl stretch relative to the C-H stretching modes [24]. CI was defined as the carbonyl group’s absorbance ratio around 1720 cm^−1^ to an internal thickness band as a reference peak at 1380 cm^−1^ [26] (CI = A_1720_/A_1380_). As the FTIR adsorption bands of TiO_2_ are located at the 439–754 cm^−1^ interval [27,28], the presence of C,N-TiO_2_ traces in the residual MPs does not affect CI calculation. The CI changes of the whole set of samples were compared with the trend in the MPs’ concentration. OM and SEM (secondary-electron imaging) were used to analyze the changes in the morphology of the MPs, again, with no interference from the presence of traces of C,N-TiO_2_. In conjunction with SEM, EDS was used to determine the incorporation of oxygen to the degraded MPs.

## 3. Results

### 3.1. HDPE MPs and C,N-TiO_2_ Characterization

**Figure 1:**
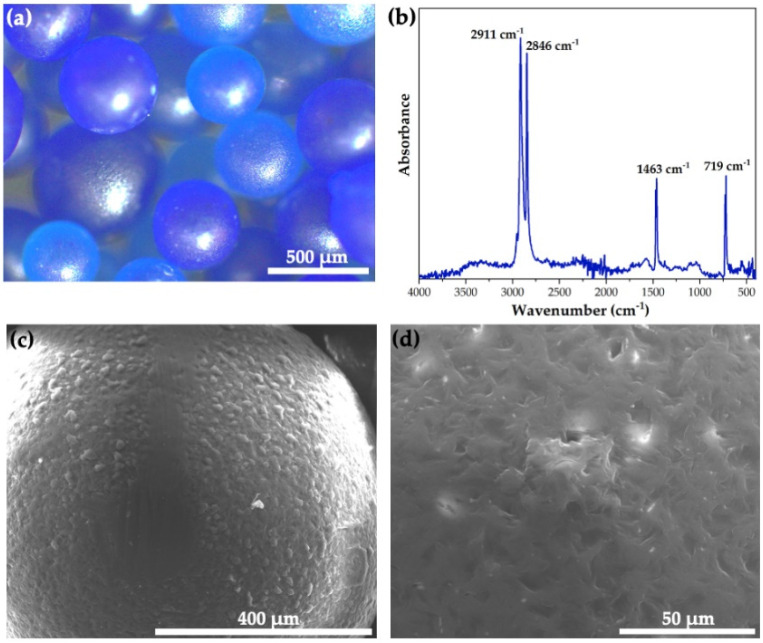
(**a**) Optical micrography, (**b**) FTIR spectrum, and (**c**,**d**) SEM micrographs of the as−extracted HDPE MPs.

As shown in Figure 1a, the blue microbeads extracted from the commercial facial scrub have an average size of 725 ± 108 μm (50 measurements). The FTIR analysis carried out on the microbeads (Figure 1b) revealed the characteristic vibrational bands of high-density polyethylene (HDPE), these being the bands at 2911 and 2846 cm^−1^ that correspond to the asymmetric and symmetric stretching vibrations of the CH_2_ group and the bands at 1463 and 719 cm^−1^ that correspond to the scissoring and rocking bending vibrations of the same group [27]. SEM micrographs demonstrated that the surface of the blue microbeads exhibited a homogeneous roughness, which may be related to their application as abrasive in facial scrubs (Figure 1c). Furthermore, some minimal roughness-related porosity was present, which leads to a surface area of 0.41 ± 0.03 m^2^/g (Figure 1d). These results confirmed that the blue microbeads extracted from the commercial facial scrub are primary MPs of HDPE.

**Figure 2:**
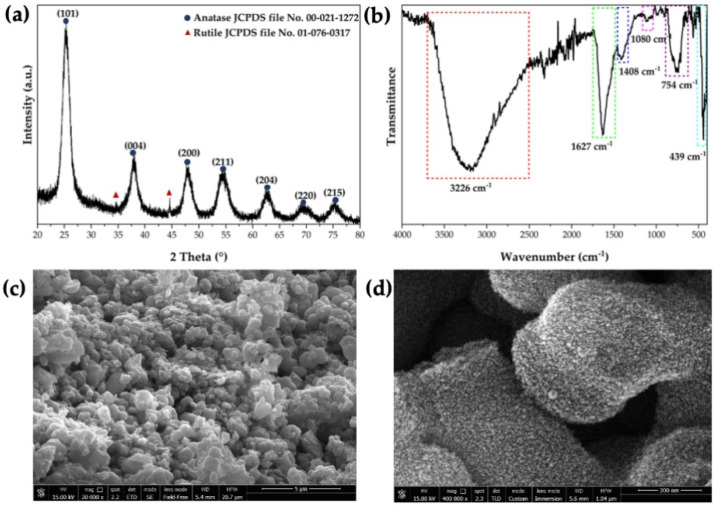
(**a**) XRD pattern, (**b**) FTIR spectrum, and (**c**,**d**) FEG−SEM micrographs of the C,N−TiO_2_ semiconductor.

For the C,N-TiO_2_ semiconductor, the XRD pattern (Figure 2a) showed that the semiconductor was mainly composed of the anatase polymorph of TiO_2_. However, two small peaks at 36° and 44° attributable to rutile TiO_2_ were also visible. Although anatase has a higher photocatalytic yield than rutile [29], it is known that the presence of rutile in mixed-phase TiO_2_ nanocomposites increases the photocatalytic activity due to the interfacial charge separation between anatase and rutile. Thus, inhibiting the recombination process of the photogenerated *h*^+^-*e*^−^ pairs [30].

On the other hand, the FTIR analysis (Figure 2b) exhibited the absorption bands corresponding to the Ti–O–Ti bonds’ stretching vibrations from the photocatalytic material, located at 439 and 754 cm^−1^ [27,28]. The broad absorption band between 3450 and 3200 cm^−1^ was assigned to the O–H bond’s stretching vibration from adsorbed water. The adsorption bands at 1408 cm^−1^ and 1080 cm^−1^ were assigned to the vibrations of the Ti–N bond [31,32], while the band located 1627 cm^−1^ is usually related to the bending vibrations of the N–H and O–H bonds [32]. The additional elemental analysis confirmed that the semiconductor contained 0.41 wt. % of nitrogen, along with 1.37 wt. % of carbon (Table 2). The presence of these elements in the semiconductor’s structure is known to promote a reduction of the bandgap of TiO_2_ from its standard value of 3.2 eV [9] to 2.90 eV (Figure S1), enabling the absorption of visible light of 428 nm (Table 2).

Figure 2c,d show the FEG-SEM micrographs of the C,N-TiO_2_ photocatalyst at different magnifications. At lower magnifications (Figure 2c), it can be observed that the semiconductor is formed by irregular particles that are ranged between 1 and 3 μm in diameter, which promoted porosity due to their agglomeration. On the other hand, higher magnification (Figure 2d) exposed that these micrometric particles are formed by smaller particles of approximately 10 nm in diameter. Thus, the microstructure of the C,N-TiO_2_ semiconductor possesses a high surface area. This was demonstrated by BET analysis, which reported that the C,N-TiO_2_ semiconductor has a surface area of 194.0 ± 1.8 m^2^/g (Table 2), being 3.88 times bigger than the surface area of commercial TiO_2_ (Aeroxide^®^ P25, 50 m^2^/g [22]). The isotherm obtained from the nitrogen absorption analysis (Figure S2) belongs to type IV, which corresponds to a mesoporous material according to the IUPAC classification (Table 2). The isotherm presented a hysteresis loop type H1, which is often associated with porous materials that consist of almost uniform agglomerated spheres [33], as observed in Figure 2d.

### 3.2. Understanding the Practical and Mechanistic Issues of Photocatalytic Degradation of MPs in an Aqueous Medium

Photocatalysis of MPs using aqueous solutions and powder semiconductors should be carefully designed as it presents some inherent practical and mechanistic issues that may negatively affect degradation. As presented in Equation (1), irradiation of the semiconductor with energy higher than its bandgap generates *h*^+^ in its valence band and *e*^−^ in the conduction band. If their recombination is avoided, *h*^+^ can oxidize organic pollutants (P) to their radical cation (Equation (2)) or react with adsorbed water or hydroxyl groups (OH^−^) to generate OH^●^ (Equations (3) and (4)), which in turn oxidize the adsorbed pollutants (Equation (5)) [9,22,34]. Photogenerated *e*^−^ reduce molecular oxygen to form O_2_^●−^ (Equation (6)), which participates in the acid–base equilibrium with its protonated form (Equation (7)) [22]. As the p*K*_a_ of HO_2_^●^ is 4.6, the acidic form of O_2_^●−^ may be the most abundant species in our reaction system (pH 3) [22,35]. Dismutation reactions of HO_2_^●^ and O_2_^●−^ generate H_2_O_2_ and O_2_ (Equations (8) and (9)) [9,22,35]. The generation of H_2_O_2_ during photocatalysis is a crucial step for further OH^●^ formation in bulk water. Once formed, H_2_O_2_ can react with the photogenerated *e*^−^ (Equation (10)), O_2_^●−^ (Equation (11)) or be decomposed by photolysis (Equation (12)) to form OH^●^ [9]. The interaction of the hydroxyl radicals with the MPs should lead to the degradation of these tiny plastic pollutants (Equation (13)). It is essential to highlight that the reaction presented in Equation (10) only produces surface-adsorbed OH^●^, while the reactions presented in Equations (11) and (12) can produce both surface-adsorbed and free OH^●^ [9]. Additionally, the direct photolysis of H_2_O_2_ under visible light irradiation can be neglected due to its small molar absorption coefficient (<1 M^−1^cm^−1^ above 300 nm) [23].

Semiconductor + *hv* ⇌ *h*^+^ + *e*^−^ (1)

*h*^+^ + P ⇌ P^●^^+^ (2)

*h*^+^ + H_2_O_ads_ → OH^●^_ads_ (3)

*h*^+^ + OH^¯^_ads_ → OH^●^_ads_ (4)

P + OH^●^_ads_ → Oxidation products (5)

*e*^−^ + O_2_ → O_2_^●−^ (*k* = 1.9 × 10^10^ M^−1^s^−1^) (6)

HO_2_^●^ ⇌ H^+^ + O_2_^●−^ (p*K*_a_ = 4.8) (7)

2 HO_2_^●^ → H_2_O_2_ + O_2_ (8)

2O_2_^●−^ + 2H^+^ → H_2_O_2_ + O_2_ (9)

H_2_O_2_ + *e*^−^ → OH^●^ + OH^−^ (10)

H_2_O_2_ + O_2_^●−^ → OH^●^ + O_2_ + OH^−^ (11)

H_2_O_2_ + *hv* → OH^●^ + OH^●^ (12)

MPs + OH^●^ → Oxidation products (13)

**Figure 3:**
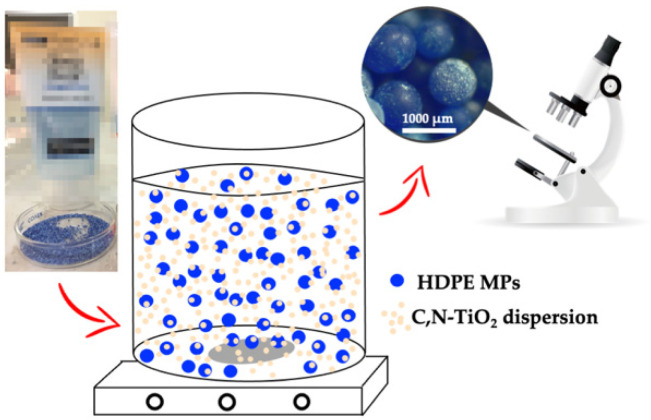
Adsorption of the C,N-TiO_2_ semiconductor in the surface of HDPE MPs.

As deduced by the previous reactions, the pollutant’s interaction with the generated reactive species (through adsorption) is crucial to the photocatalytic process. When performing heterogeneous photocatalysis in an aqueous medium, the interaction between the pollutant and the semiconductor is usually achieved by the adsorption of the former at the surface of the photocatalyst. However, MPs pollutants cannot be adsorbed at the surface of the photocatalyst. As presented in Figure 3, the size of HDPE MPs limits their interaction with the powder semiconductor.

The MPs used in this work are nearly 241-fold the size of C,N-TiO_2_ biggest particles, precluding their adsorption on the semiconductor and limiting their oxidation by *h*^+^ and adsorbed OH^●^. However, degradation may still be promoted whether the semiconductor is used in the form of a powder dispersion. As shown in Figure 3, the C,N-TiO_2_ semiconductor forms a coating on the HDPE MPs’ surface [13]. Nonetheless, the MPs’ coverage with C,N-TiO_2_ may be limited by the low porosity and surface area of the HDPE microbeads.

### 3.3. Effect of Free OH^●^ on HDPE MPs Photocatalytic Degradation

The interaction between the HDPE MPs and the C,N-TiO_2_ powder semiconductor is limited when photocatalysis is performed in an aqueous medium. Thus, it is very likely that most of the photocatalytic degradation of MPs is mainly conducted by the free OH^●^ radicals present in the bulk of the medium. To determine the free OH^●^ contribution in HDPE MPs degradation, photocatalysis was carried out in the absence and presence of tert-butanol as OH^●^ scavenger.

**Figure 4:**
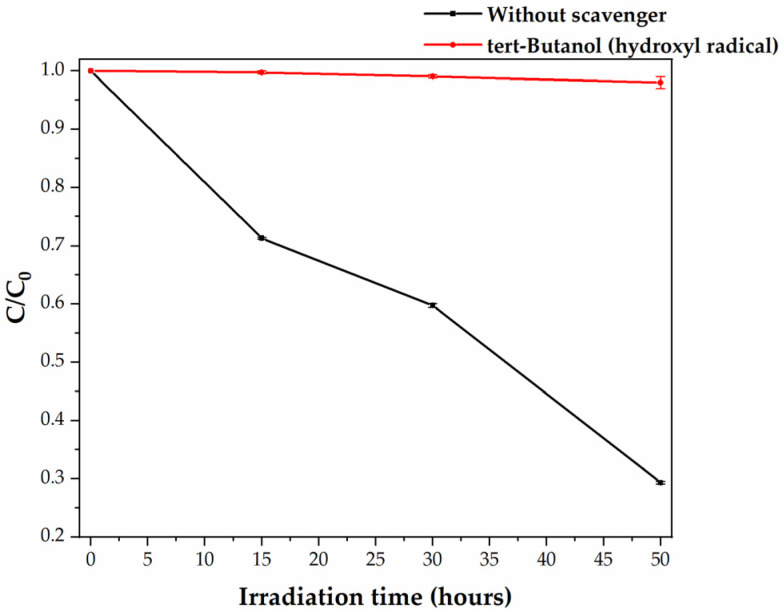
Visible-light photocatalytic degradation of HDPE MPs by C,N-TiO_2_ in the absence and presence of tert-Butanol as free OH^●^ scavenger.

Figure 4 presents the degradation plot for the visible-light photocatalysis of HDPE MPs by C,N-TiO_2_ in the absence and presence of tert-butanol. The black curve shows that without scavenger, i.e., when free OH^●^ are present in the reaction system, the MPs’ concentration was reduced by 71.77%. As previously reported, degradation at these conditions was related to the increase of the MPs’ surface area due to their fragmentation at 0 °C and the effect of acidic pH in the plastic degradation mechanism and the semiconductor deagglomeration [13]. The red curve of Figure 4 shows that the inhibition of the reaction between HDPE MPs and free OH^●^ (*k* for the tert-butanol/OH^●^ reaction is 6.2 × 10^8^ M^−1^s^−1^, Table 1) dramatically reduces the degradation of MPs. The MPs concentration reduction passed from 71.77% at normal conditions to 1.98% when tert-butanol is present in the reaction system. This behavior indicates that free OH^●^-mediated oxidation processes are the predominant pathways leading to the HDPE MPs’ degradation. This result is in good agreement with that obtained by Jiang et al. [11] and with the LDPE and PP MPs degradation pathways suggested by the research group of J. Dutta [10,36]. For this work, the reaction systems characteristics discussed in Section 3.2 suggest that oxidation of HDPE MPs by C,N-TiO_2_ is promoted OH^●^ formation through the reaction pathways involving the photogenerated *e*^−^ (Equations (6)–(11)). The fact that some degradation was still detected at these reaction conditions was related to the deposition of the C,N-TiO_2_ powders on the MPs’ surface (Figure 3), a phenomenon that may facilitate the direct oxidation of the HDPE microbeads with the photogenerated *h*^+^ (Equation (2)). To test this hypothesis, photocatalytic experiments using IPA as an *h*^+^ scavenger were performed.

### 3.4. Effect of h^+^ on HDPE MPs Photocatalytic Degradation

Holes are the primary oxidizing species in photocatalytic reactions [9]. Although they are not usually related to plastic degradation by photocatalysis, photogenerated *h*^+^ can carry out the direct oxidation of organics when these compounds can act as electron donors [9,37]. For example, Ueckert et al. reported that photoreforming of poly(ethylene terephthalate) (PET) and poly(lactic acid) (PLA) could be carried out by carbon nitride/nickel phosphide (CN_x_|Ni_2_P) photocatalyst [38]. By performing this process in the presence of terephthalic acid (TPA) as an OH^●^ scavenger, authors found that OH^●^ plays a minimal role in the oxidation of plastics and that photoreforming instead proceeds via direct *h*^+^ transfer between the catalyst and the substrate [38]. Here, to determine the contribution of *h*^+^ in the degradation of HDPE MPs, photocatalysis was carried out in the presence of IPA as an *h*^+^ scavenger [22]. As tert-butanol, IPA can also act as OH^●^ scavenger (*k* = 2 × 10^9^ M^−1^s^−1^, Table 1). However, due to its smaller size, IPA presents strong dissociative and non-dissociative chemisorption on TiO_2_ [39]. Thus, adsorbed IPA acts as an electron donor and is oxidized by *h*^+^.

**Figure 5:**
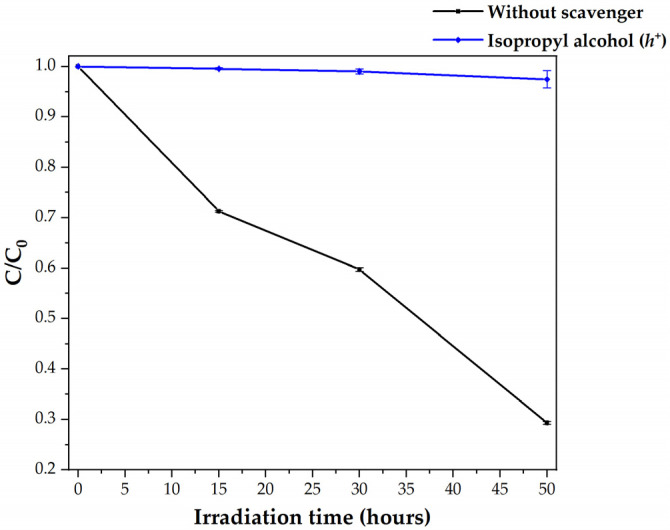
Visible-light photocatalytic degradation of HDPE MPs by C,N-TiO_2_ in the absence and presence of IPA as *h*^+^ and OH^●^ scavenger.

Figure 5 presents the degradation plot for the visible-light photocatalysis of HDPE MPs by C,N-TiO_2_ in the absence and presence of IPA.

The blue curve of Figure 5 shows that when IPA is added to the reaction system, MPs’ photocatalytic removal is inhibited. IPA decreases MPs degradation by adsorbing on the semiconductor surface, reacting with the photogenerated *h*^+^, and avoiding Reaction 2. As IPA also eliminates OH^●^ from the reaction system, this result suggests that HDPE MPs are oxidized by the free OH^●^ and directly by the photogenerated *h*^+^. These species can react with the free electron-containing ending groups of the PE chain [40] through the photo-Kolbe reaction [41] (Equation (14)).

RCOO¯ + *h*^+^ → R^●^ + CO_2_ (14)

At these reaction conditions (similar to those reported in Section 3.3 for tert-butanol scavenger), low degradation of the HDPE MPs was detected (HDPE concentration was reduced by 2.53%). This result was related to the deposition of the C,N-TiO_2_ powders on the MPs’ surface, a phenomenon that in this system (without *h*^+^ and OH^●^) may facilitate the HDPE oxidation microbeads with O_2_^●−^. As recombination of the photogenerated *h*^+^-*e*^−^ pairs was avoided by scavenging *h*^+^ with IPA, the reaction presented in Equation (6) is favored, and O_2_^●−^ is expected to be present in the reaction system. As OH^●^, O_2_^●−^ is often believed to play an essential role in initiating the polyethylene degradation process by attacking the polymeric chain [17,26]. To test this hypothesis, photocatalytic experiments using Tiron as an O_2_^●−^ scavenger were performed.

### 3.5. Effect of O_2_^●−^ on HDPE MPs Photocatalytic Degradation

The role of O_2_^●−^ on the photocatalytic degradation organics in water is frequently evaluated using p-benzoquinone scavenger (*k* for p-benzoquinone/O_2_^●−^ reaction is 0.9–1 × 10^9^ M^−1^s^−1^) [22]. However, p-benzoquinone does not only react with O_2_^●−^, but also with *e*^−^ (*k* = 1.35 × 10^9^ M^−1^s^−1^) and OH^●^ (*k* = 6.6 × 10^9^ M^−1^s^−1^) [22]. The use of p-benzoquinone as an O_2_^●−^ scavenger has other drawbacks. When photoexcited with wavelengths above 310 nm, p-benzoquinone produces singlet oxygen (^1^O_2_) [42], which may contribute to the oxidation of organic molecules [9]. On the other hand, Garg et al. have postulated that quinones in natural organic matter are subjected to photolysis under sunlight, generating O_2_^●−^, which further evolves to H_2_O_2_ and OH^●^ [43]. Tiron was chosen as O_2_^●−^ scavenger (*k* for Tiron/O_2_^●−^ reaction is 5 × 10^8^ M^−1^s^−1^, Table 1) due to the previous motivations.

**Figure 6:**
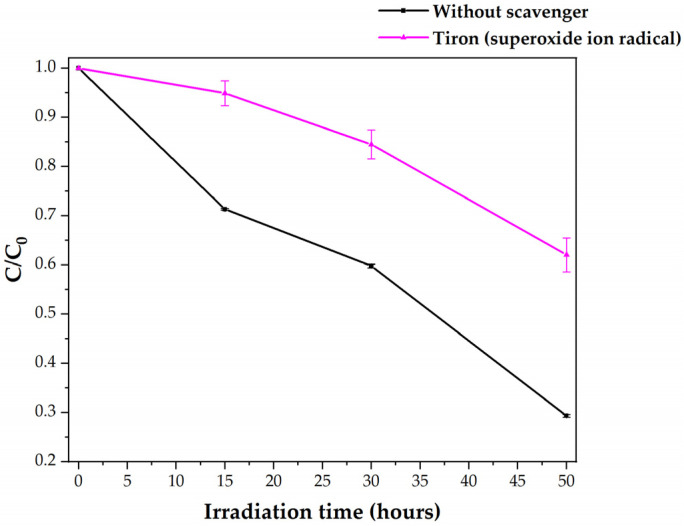
Visible-light photocatalytic degradation of HDPE MPs by C,N-TiO_2_ in the absence and presence of Tiron as O_2_^●^^−^ scavenger.

Figure 6 presents the degradation plot for the visible-light photocatalysis of HDPE MPs by C,N-TiO_2_ in the absence and presence of Tiron. The pink curve of Figure 6 shows that when Tiron is added to the reaction system, MPs’ photocatalytic removal is reduced but not completely inhibited (MPs’ concentration was reduced by 37.98%). This inhibition is in good agreement with the previously proposed photocatalytic degradation pathways of LDPE and PP MPs [10,36]. To explain the partial inhibition of photocatalysis, three phenomena should be taken into account: The absence of (i) O_2_^●−^ and (ii) O_2_^●−^-derived OH^●^ in the reaction system and (iii) the presence of adsorbed *h*^+^ on the C,N-TiO_2_ surface. First, some reports indicate that O_2_^●−^ is involved in the direct oxidations of organic molecules that can act as a proton source [9,44], as O_2_^●¯^ can react with a proton donor to form HO_2_^●^ (Equation (15)) [44]:

O_2_^●−^ + HX ⇌ HO_2_^●^ + X^−^ (15)

**Scheme 1:**
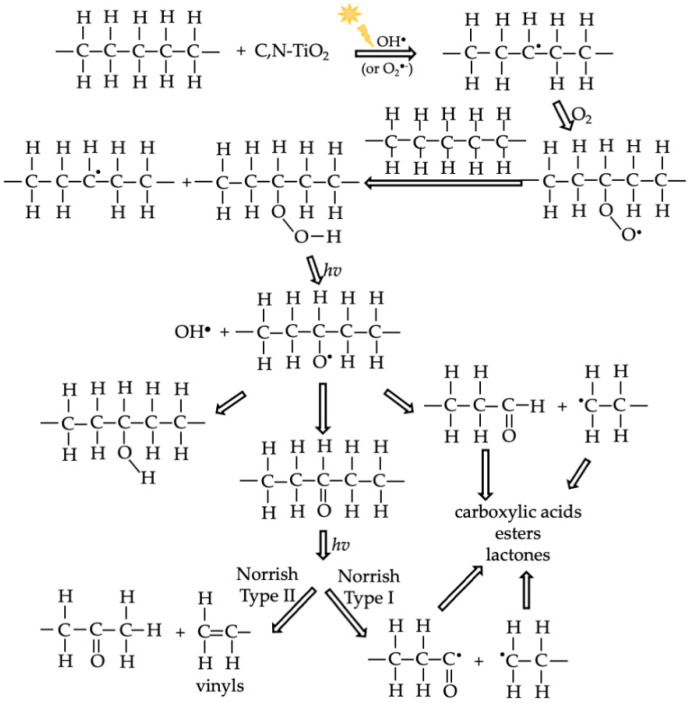
Proposed mechanism for HDPE MPs photocatalytic degradation with C,N-TiO_2_.

Here, O_2_^●−^ may promote the initiation step of PE oxidation by the abstraction of a proton of the polymeric chain (see Scheme 1 in Section 3.7). Therefore, degradation is in part inhibited when this species is not present in the reaction system. However, when O_2_^●−^ reacts with Tiron, the acid-based equilibrium presented in Equation (7) is perturbed. As a consequence, the formation of H_2_O_2_ through Equations (8) and (9) is avoided. As H_2_O_2_ further evolves to OH^●^ through Equations (10) and (11), this specie is not expected to be present in this particular reaction system. As the role of the OH^●^ in the MPs degradation has been ruled out in Section 3.3, the decrease in degradation is explained by its absence from the reaction system. Consequently, this suggests that O_2_^●−^ plays an essential role in HDPE MPs photocatalytic degradation by extracting protons from the PE chain and their conversion to H_2_O_2_ and OH^●^. Lastly, the reduction of the HDPE degradation (instead of the complete inhibition) of Figure 6 confirms MPs’ oxidation by the adsorbed *h*^+^ species through Reaction 2. As ^1^O_2_ cannot be present in the reaction system because it is formed through oxidation of the absent O_2_^●−^ by the adsorbed *h*^+^ [9], the only plausible path for MPs degradation in the presence of Tiron is the direct oxidation by *h*^+^. The lower degradation of MPs concerning the experiment without scavenger (black curve) was related to the limited deposition of C,N-TiO_2_ powders on the MPs. The crucial role of the O_2_^●−^ in the photocatalytic degradation of HDPE MPs presented here differs from the result obtained by Juang et al. [11], where the absence of this reactive species in their system did not affect the degradation of HDPE MPs by BiOCl photocatalyst. This difference can be related to the use of p-benzoquinone as an O_2_^●−^ scavenger [11], which can lead to the formation of ^1^O_2_ [42], H_2_O_2_ [43] and OH^●^ [43] in the reaction system. From these, OH^●^ was observed to be the main specie that promotes degradation [11].

### 3.6. Effect of e^−^ on HDPE MPs Photocatalytic Degradation

**Figure 7:**
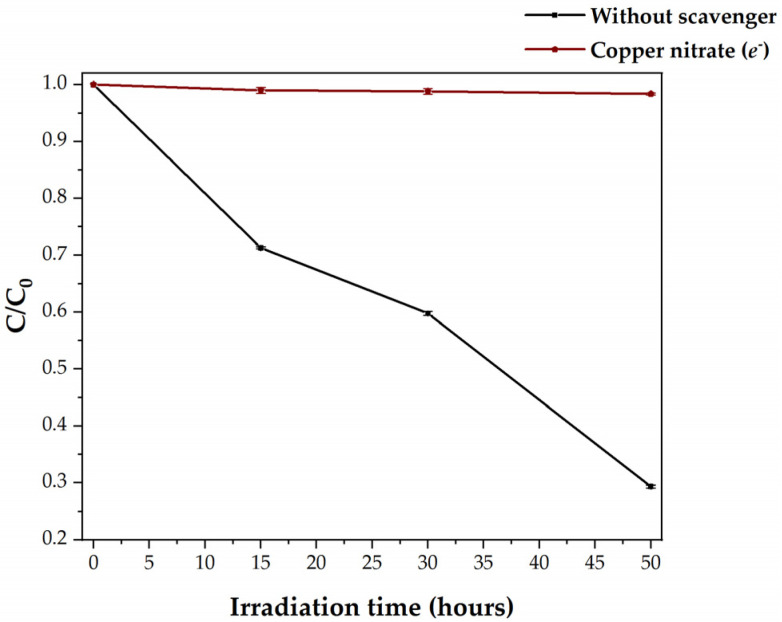
Visible-light photocatalytic degradation of HDPE MPs by C,N-TiO^2^ in the absence and presence of copper nitrate as an *e*^−^ scavenger.

Copper nitrate was used to determine the role of the photogenerated *e*^−^ in the HDPE degradation. This reagent was used because the adsorption of the NO_3_^−^ anions on the surface of TiO_2_ is known to be weak [23]. Figure 7 presents the degradation plot for the visible-light photocatalysis of HDPE MPs by C,N-TiO_2_ in the absence and presence of Cu(NO_3_)_2_.

As observed in the bordeaux colour curve of Figure 7, the presence of copper nitrate in the reaction system inhibits MPs degradation. This result can be explained by analyzing the critical steps of the photocatalytic mechanism where photogenerated *e*^−^ participates. As Cu^2+^ cation adsorbs in the surface of C,N-TiO_2_, it reacts with the *e*^−^, inhibiting the formation of O_2_^●−^ through the reaction presented in Equation (6) [23]. As explained previously, the absence of O_2_^●−^ inhibits the generation of H_2_O_2_ and its further conversion into free OH^●^ (Equations (6)–(9)). Furthermore, removing *e*^−^ from the reaction system inhibits the reduction of H_2_O_2_ to OH^●^ (Equation (10)). This latter is a key element for the degradation of HDPE MPs by photocatalysis with C,N-TiO_2_. Since the quenching of *e*^−^ with copper nitrate avoids *h*^+^-*e*^−^ recombination, the low degradation of the MPs at these conditions (1.64%) was attributed to the adsorbed *h*^+^ and their interaction with the MPs.

**Figure 8:**
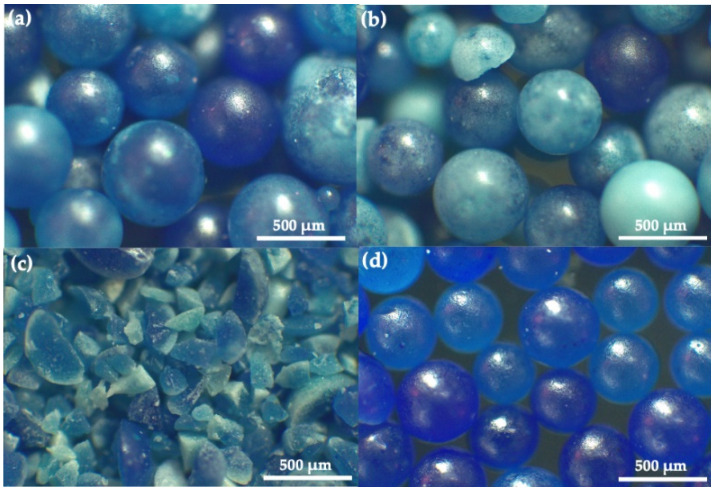
Optical micrographs of the HDPE MPs after visible-light photocatalytic degradation for 50 h by C,N-TiO_2_ in the presence of (**a**) tert-Butanol, (**b**) isopropyl alcohol (**c**) Tiron, and (**d**) copper nitrate scavengers.

Figure 8 presents the optical micrographs of the HDPE MPs after visible-light photocatalytic degradation by C,N-TiO_2_ in the four scavengers’ presence. The MPs subjected to photocatalysis in reaction media without OH^●^, *h*^+^, and *e*^−^ show the same morphology as the as-extracted microplastics of Figure 2a. On the other hand, the photocatalytically degraded MPs in the absence of O_2_^●−^ are highly disintegrated (Figure 8c), confirming plastic degradation [45].

**Figure 9:**
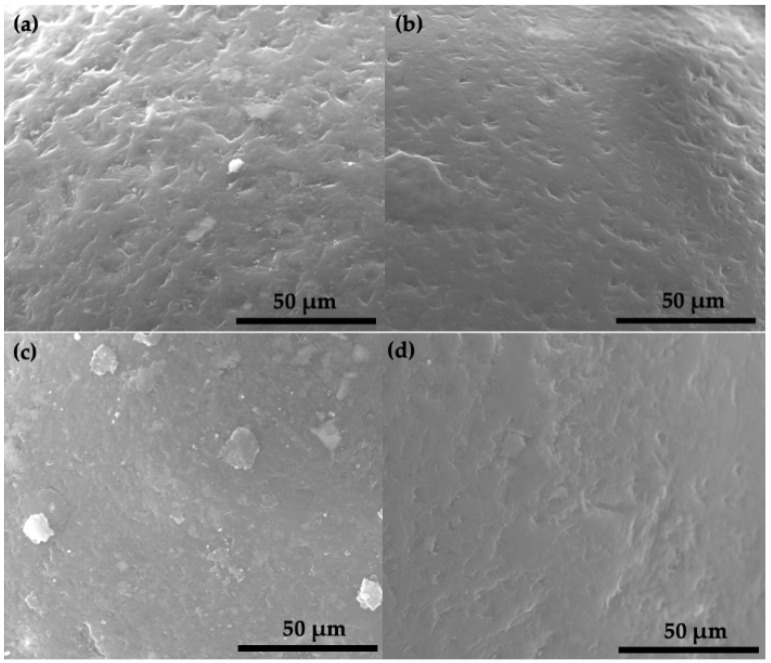
SEM micrographs of the HDPE MPs after visible-light photocatalytic degradation for 50 h by C,N-TiO_2_ in the (**a**) tert-Butanol, (**b**) isopropyl alcohol (**c**) tiron, and (**d**) copper nitrate scavengers.

A deeper insight into the changes of the MPs morphology was determined by SEM (Figure 9). The SEM micrographs of the microbeads subjected to photocatalysis without OH^●^, *h*^+,^ and *e*^−^ show the same rough surface as the as-extracted microplastics of Figure 2d, while the MPs that were degraded in the absence of O_2_^●−^ present a slightly smoother surface. The loss of roughness was related to the degradation of the more external surface of the HDPE MPs by the adsorbed *h*^+^, as most plastics degrade first at the polymer surface because it is exposed and available for the chemical attack [13,45].

EDS chemical microanalysis of the as-extracted and degraded MPs are presented in Figures S3 and S4, respectively. In both cases, the signal from gold corresponds to the conductive layer deposited during sample preparation. For the as-extracted MPs, the EDS spectra were taken from several microbeads and they present the intense signal of carbon from polyethylene polymer. As observed in Figure S3, the oxygen signal is practically absent from this sample, indicating that the as-extracted MPs are not oxidated. The EDS spectra from Figure S4 correspond to the MPs subjected to photocatalysis for 50 h at pH 3 and 0 °C, without scavengers. The oxygen signal is present in all the spectra. However, the EDS analysis interpretation must take into account both oxidation and the presence of C,N-TiO_2_ on the plastic’s surface, as both contribute to the oxygen signal. Figure S5 presents a higher magnification micrograph of the same sample, where the photocatalyst residues are visible. By taking into account the stoichiometry of TiO_2_, the EDS analysis presented in Table S1 demonstrates that the presence of oxygen in this sample can be related to plastic oxidation (spectrum 6), the semiconductor (spectra 1 and 2) or a combination of both (spectra 3–5).

The calculation of CI from the FTIR spectra (Figure S6) also confirmed the degradation of plastics. As observed in Table 3, the as-extracted MPs present a CI value of 0.86, which was related to chromophores’ presence [26,36]. When photocatalysis is carried out without scavengers, MPs degradation led to an increase in the CI of 29%. The CI values of the microbeads subjected to photocatalysis without OH^●^, *h*^+^, and *e*^−^ are similar to that of the as-extracted microplastics, while the MPs degraded in the absence of O_2_^●−^ present an increase of 19% with respect to the pristine MPs. This intermediate value between the original and the most degraded MPs is in good agreement with reducing the HDPE concentration observed for this particular reaction system.

### 3.7. Proposed Mechanism for HDPE MPs Photocatalytic Degradation

The proposed mechanism for PE degradation is presented in Scheme 1.

As previously reported in the literature, in the initiation step the OH^●^ break the C–H bonds of the polymer backbone to produce polyethylene alkyl radicals [36,45]. The results suggest that the initiation step can also be promoted by the abstract of a hydrogen atom from the polymeric chain by O_2_^●−^. This reaction’s product is HO_2_^●^, which form more OH^●^ by the dismutation reaction presented in Equation (8). Moreover, the photo-Kolbe type reaction between the free electron-containing ending groups of the PE chain and the *h*^+^ may also generate polyethylene alkyl radicals [41]. During propagation, the alkyl radicals react with O_2_ to generate peroxy radicals (–CH_2_-CH_2_–HCOO^●^–CH_2_-CH_2_-), which form the hydroperoxide species (–CH_2_-CH_2_–HCOOH–CH_2_-CH_2_-) by extracting a hydrogen atom from another polymer chain. This chemical specie is further divided into two new free oxy and OH^●^ radicals by the scission of the weak O–O bond [46,47]. After the formation of hydroperoxides, complex radical reactions take place and lead to autoxidation. The propagation step leads to chain scission or crosslinking, promoting a decrease or increase in molecular weight. Finally, termination of the radical reaction takes place when the combination of two radicals forms inert products such as alkenes, aldehydes, and ketones.

The results obtained here confirm that the photocatalysis of MPs is a complex phenomenon that strongly depends on the reaction system’s particular characteristics. Due to their bigger size (relative to C,N-TiO_2_ particles), MPs do not adsorb on the semiconductor. Instead, the C,N-TiO_2_ powders adsorb on the plastic’s surface. This phenomenon limits the interaction between MPs, *h*^+^ and OH^●^_ads_. Despite this drawback, MPs’ oxidation is still possible thanks to the formation of O_2_^●−^ and free OH^●^ (Reactions 6 and 11). Since both species are generated from the reaction pathways involving the photogenerated *e*^−^, the results presented in this work reveal for the first time that the photogenerated *e*^−^ plays an essential role in the photocatalytic degradation of HDPE MPs. Furthermore, the degradation behaviors observed when *h*^+^ and O_2_^●−^ were absent from the reaction system suggest that the initiating step of MPs degradation can be performed by OH^●^ and the photogenerated *h*^+^ or O_2_^●−^.

## 4. Conclusions

In this work, an in-depth analysis regarding the participation of OH^●^, *h*^+^, O_2_^●−^ and *e*^−^ in the photocatalytic degradation of primary HDPE MPs by C,N-TiO_2_ powders was performed using scavengers. The information presented in this work can contribute to the proper design of effective photocatalytic reaction systems that enhance the degradation of HDPE MPs. As the species adsorbed in the photocatalyst’s surface have less contribution to degradation due to their limited interaction with the microbeads, degradation should be mainly based on the generation of free OH^●^ in the bulk of the solution. This can be achieved, for example, by using photocatalysts specifically designed for avoiding *h*^+^-*e*^−^ recombination and well-oxygenated reaction mediums that enhance the OH^●^ generation from the *e*^−^-derived reaction pathways. Additionally, photocatalysis can be performed in solar light to further promote the generation of free OH^●^ from the photolysis of H_2_O_2_.

## Figures and Tables

**Table 1:** Chemical reagents used as OH^●^, *h*^+^, O_2_^●−^ and *e*^−^ scavengers.

Reagent	Scavenged Specie	*k* (M^−1^s^−1^) ^a^	Reference
Tert-butanol	OH^●^ in bulk water	6.2 × 10^8^	[22]
IPA	*h* ^+^	NA	[22]
	OH^●^	2 × 10^9^	[22]
Tiron	O_2_^●^^−^	5 × 10^8^	[22]
Copper nitrate	*e^−^*	NA	[23]

^a^ Rate constant of the reaction between the reagent and the scavenged species.

**Table 2:** Properties of the C,N-TiO_2_ semiconductor.

Property	Value
Carbon content (wt. %)	1.37
Nitrogen content (wt. %)	0.41
E_g_ (eV)	2.90
Wavelength of light absorption (nm)	428
BET surface area (m^2^/g)	194.0 ± 1.8
Porosity type	Mesoporous

**Table 3:** Comparison of the CI of the as-extracted HDPE MPs with those subjected to visible-light photocatalytic degradation by C,N-TiO_2_ in the presence of scavengers.

HDPE MPs	MPs’ Degradation (%)	CI (A_1720_/A_1380_)
As-extracted	-	0.86
Without scavengers	71.77 ± 1.88 [13]	1.11
With OH^●^ scavenger	1.98 ± 0.60	0.85
With *h*^+^ scavenger	2.53 ± 1.00	0.80
With O_2_^●^**^−^** scavenger	37.98 ± 1.98	1.03
With *e^−^* scavenger	1.62 ± 0.10	0.70

## Data Availability

The data presented in this study are available on request from the corresponding authors.

## Supplementary Materials

The following are available online at https://www.mdpi.com/2073-4360/13/7/999/s1, Figure S1: (a) Reflectance spectra and (b) Plot of (F(R)**hν*)^1/2^ vs. *hν* of the C,N-TiO_2_ photocatalyst. Figure S2. N_2_ adsorption–desorption isotherms of the C,N-TiO_2_ photocatalyst. Figure S3. SEM-EDS analysis of the as-extracted HDPE MPs. Figure S4 SEM-EDS analysis of the HDPE MPs after photocatalysis at 0 °C and pH 3 for 50 h. Figure S5. SEM micrograph of the HDPE MPs after photocatalysis at 0 °C and pH 3 for 50 h (high magnification). Table S1. EDS chemical microanalysis of the HDPE MPs after photocatalysis at 0 °C and pH 3 for 50 h. Figure S6. FTIR spectra of the HDPE MPs before and after 50 h of photocatalytic degradation with and without scavengers.
