# Supplementary material for: The Role of the Reactive Species Involved in the Photocatalytic Degradation of HDPE Microplastics Using C,N-TiO2 Powders
Source: Polymers (Basel). 2021 Mar 24;13(7):999. doi: 10.3390/polym13070999 (PMC8037624; doi:10.3390/polym13070999)
Supplement: Supplementary file 1 [file polymers-13-00999-s001.pdf]

Supplementary Materials

The role of the reactive species involved in the photocatalytic degradation of HDPE microplastics using C,N-TiO$_2$ powders

Aranza Denisse Vital-Grappin [1], Maria Camila Ariza-Tarazona [2], Valeria Montserrat Luna-Hernández [1], Juan Francisco Villarreal-Chiu [1,3,*], Juan Manuel Hernández-López [1], Cristina Siligardi [2], Erika Iveth Cedillo-González [1,2,*]

[1]Universidad Autónoma de Nuevo León, Facultad de Ciencias Químicas. Av. Universidad S/N Ciudad Universitaria, San Nicolás de los Garza C.P. 66455, Nuevo León, México.

[2]Department of Engineering "Enzo Ferrari", University of Modena and Reggio Emilia, Via P. Vivarelli 10/1, 41125 Modena, Italy.

[3]Centro de Investigación en Biotecnología y Nanotecnología (CIByN), Facultad de Ciencias Químicas, Universidad Autónoma de Nuevo León. Parque de Investigación e Innovación Tecnológica, Km. 10 autopista al Aeropuerto Internacional Mariano Escobedo, Apodaca 66629, Nuevo León, México

*Correspondence: ecedillo@unimore.it (E.I.C.-G.) and juan.villarrealch@uanl.edu.mx (J.F.V.-C.)

Figure S1a presents the reflectance spectra of the of C,N-TiO$_2$ photocatalyst.

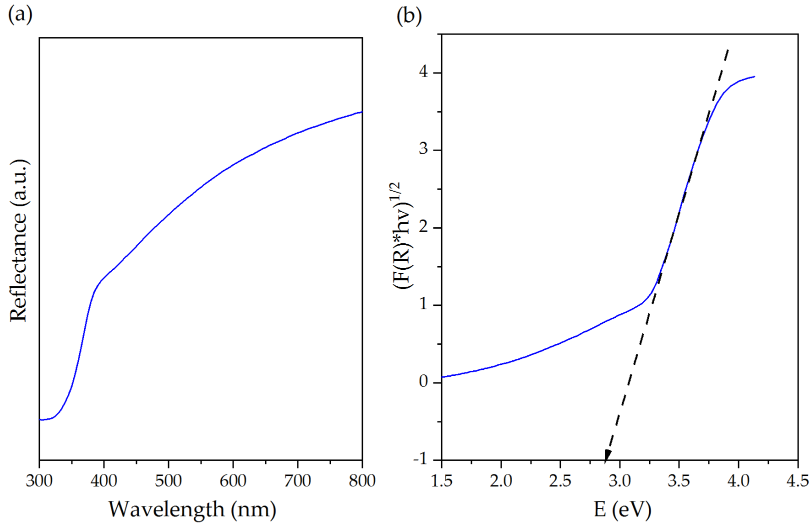

Figure S1. (a) Reflectance spectra and (b) Plot of $(F(R)*h\nu)^{1/2}$ vs $h\nu$ of the C,N-TiO$_2$ photocatalyst.

The bandgap (E_g) was determined using the Kubelka-Munk model:

$$F(R) = (1\text{-}R)^2/2R \qquad \text{(Equation S1)}$$

where F(R) is the reflectance transformed according to Kubelka-Munk theory and R is the reflectance. The bandgap value was obtained by extrapolating the linear portion of a plot of $[F(R)hv]^{1/2}$ *vs* E in eV to $[F(R)hv]^{1/2} = 0$ (Figure S1b).

Figure S2 presents the nitrogen adsorption-desorption isotherm of the photocatalyst.

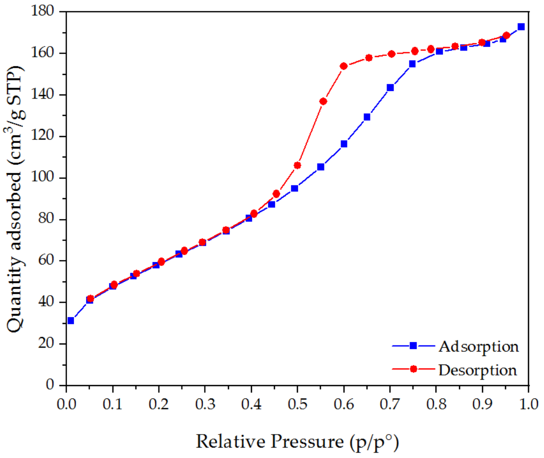

Figure S2. N_2 adsorption-desorption isotherms of the C,N-TiO_2 photocatalyst.

Figure S3 shows the SEM-EDS analysis of the as-extracted HDPE MPs.

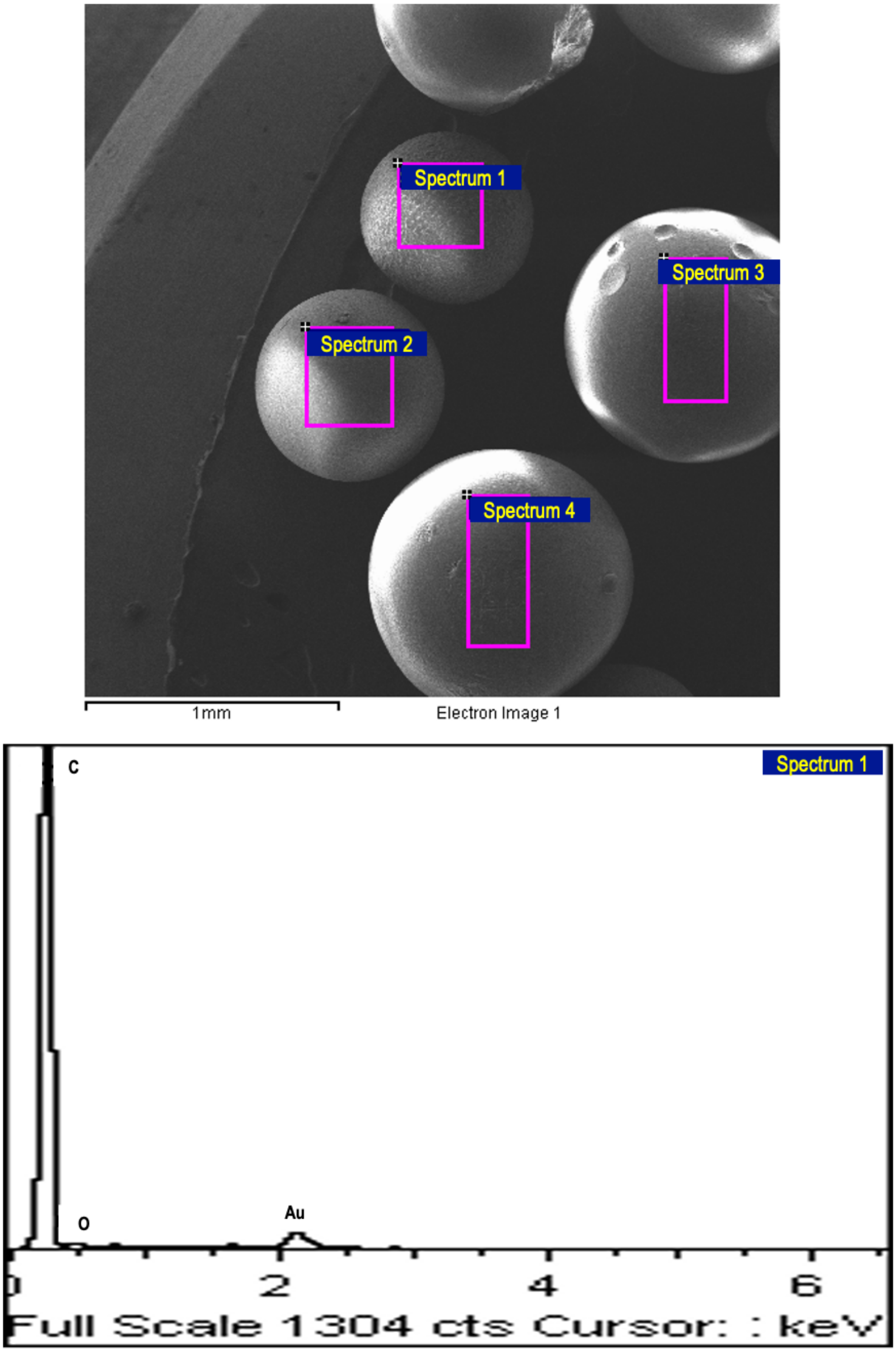

Figure S3. SEM-EDS analysis of the as-extracted HDPE MPs.

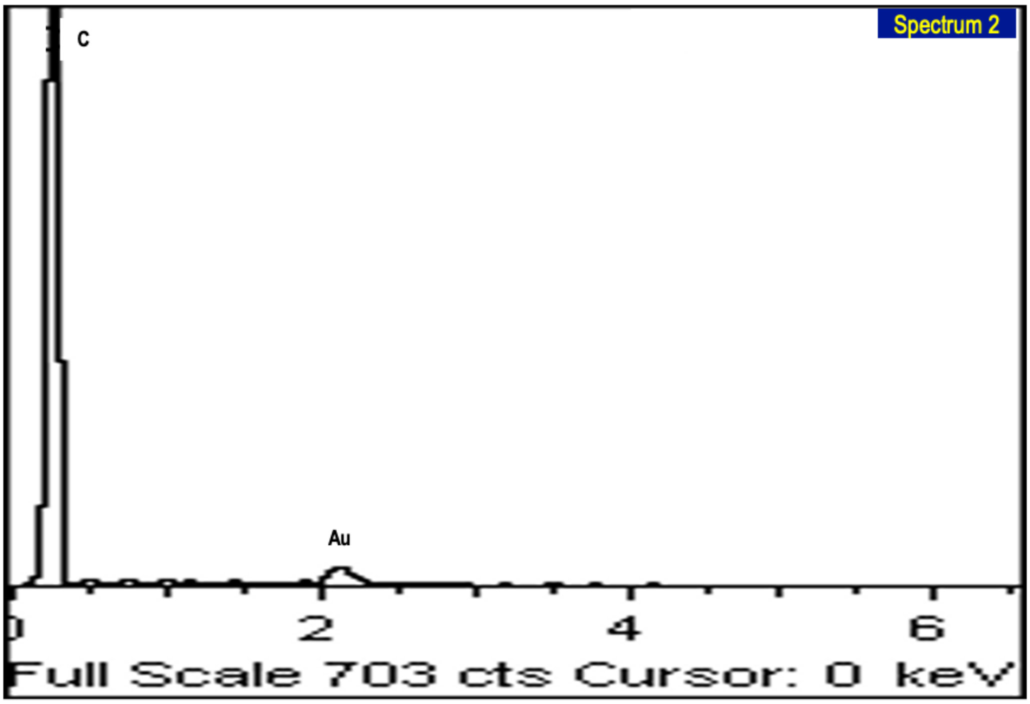

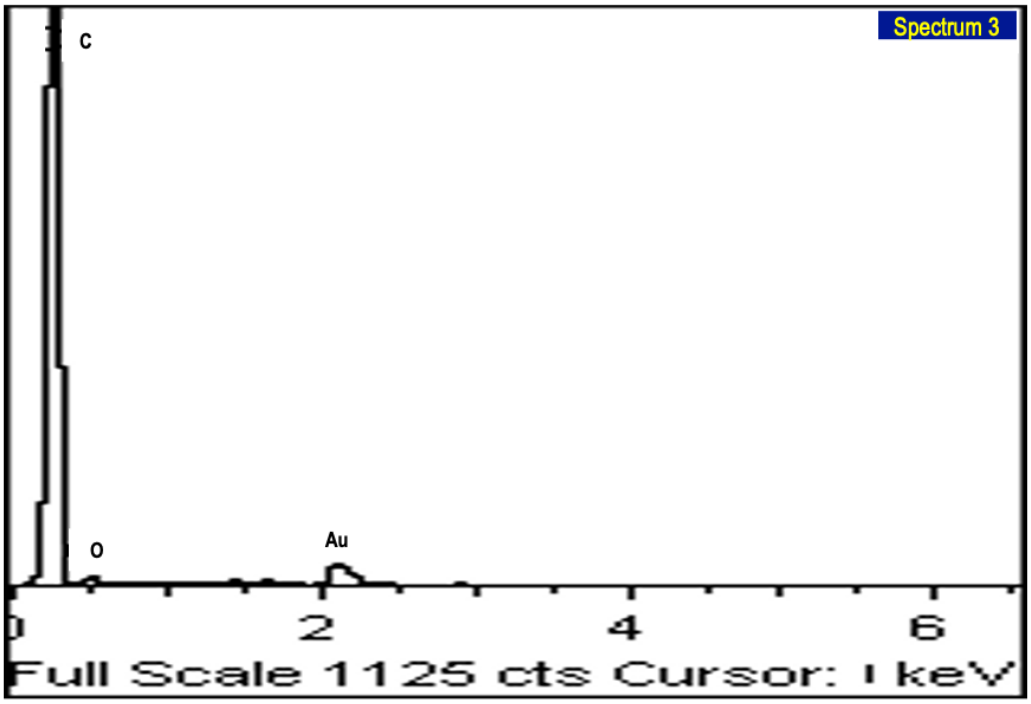

Figure S3. SEM-EDS analysis of the as-extracted HDPE MPs (continued).

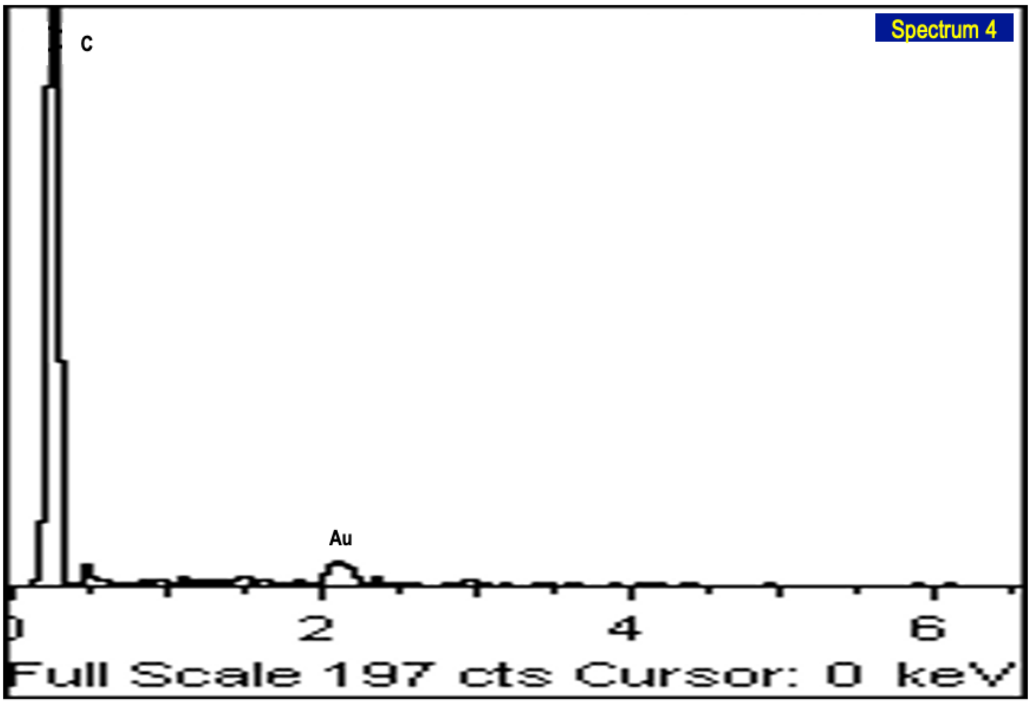

Figure S3. SEM-EDS analysis of the as-extracted HDPE MPs (continued).

Figure S4 presents the SEM-EDS analysis of the HDPE MPs after photocatalysis at 0 °C and pH 3 for 50 hours (without scavengers).

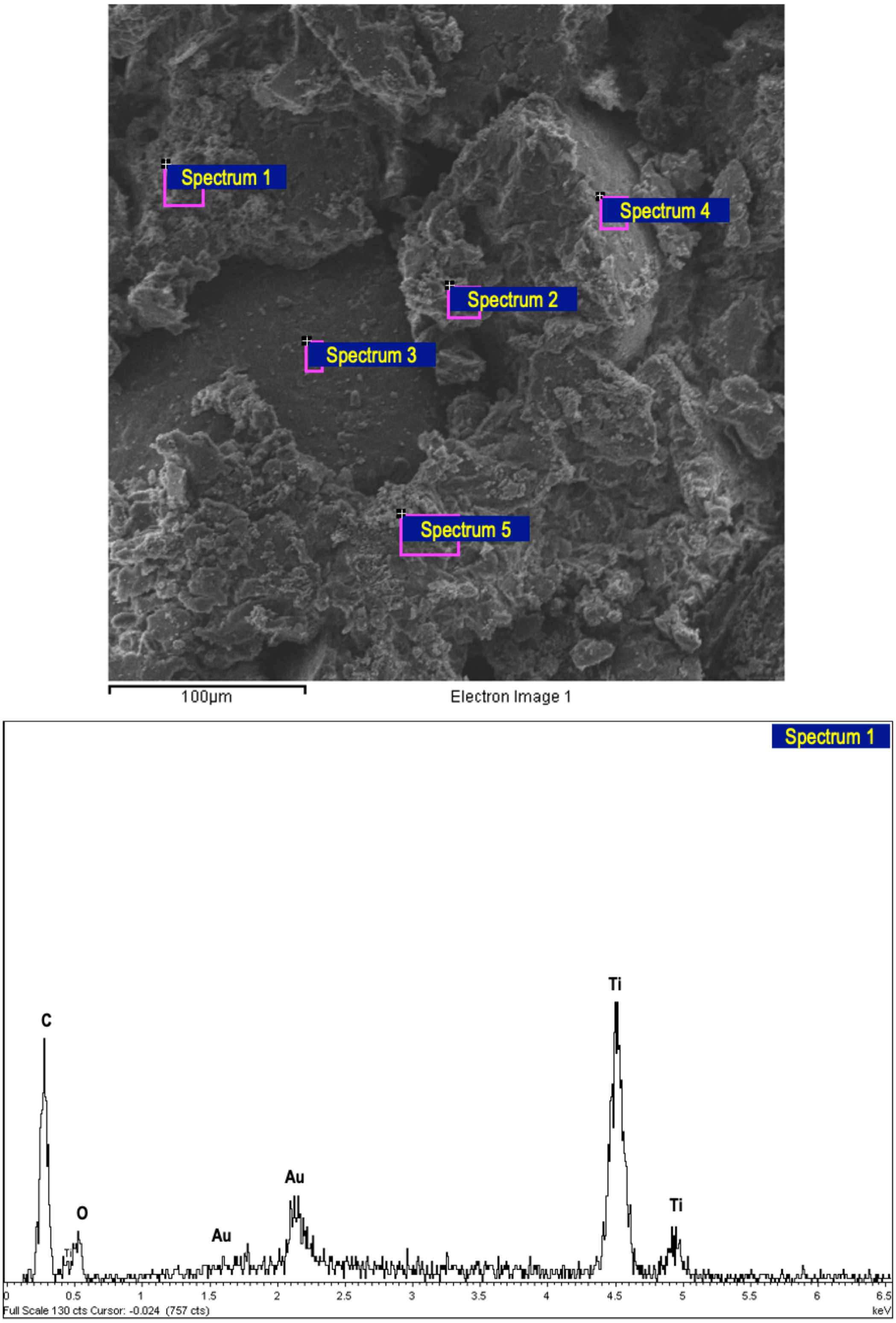

Figure S4. SEM-EDS analysis of the HDPE MPs after photocatalysis at 0 °C and pH 3 for 50 hours.

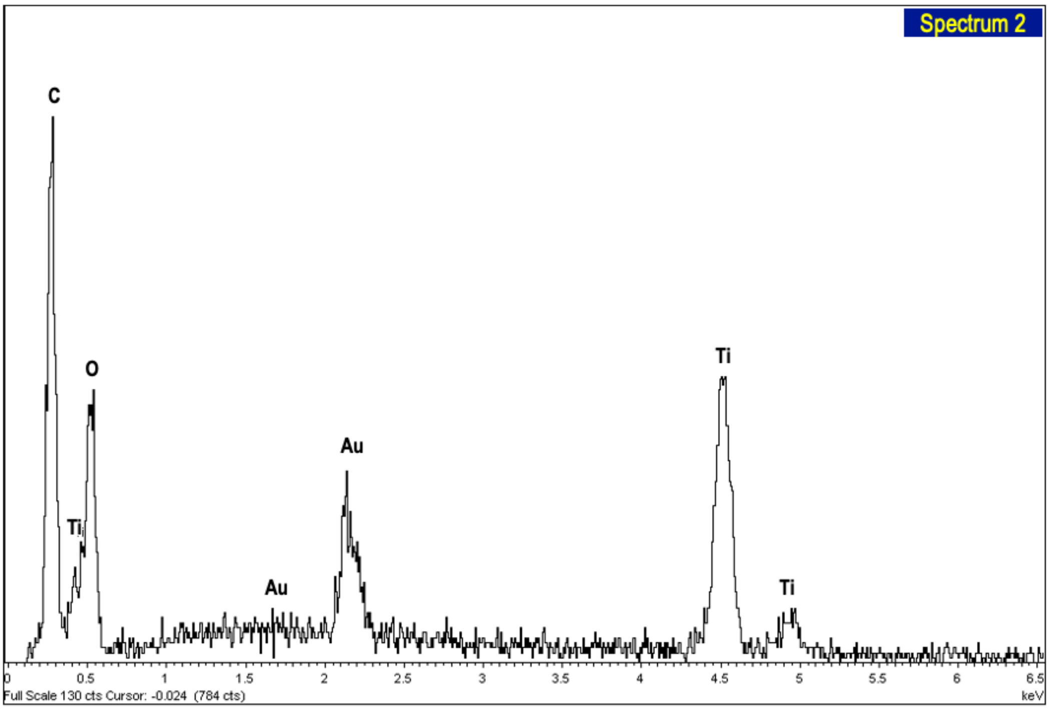

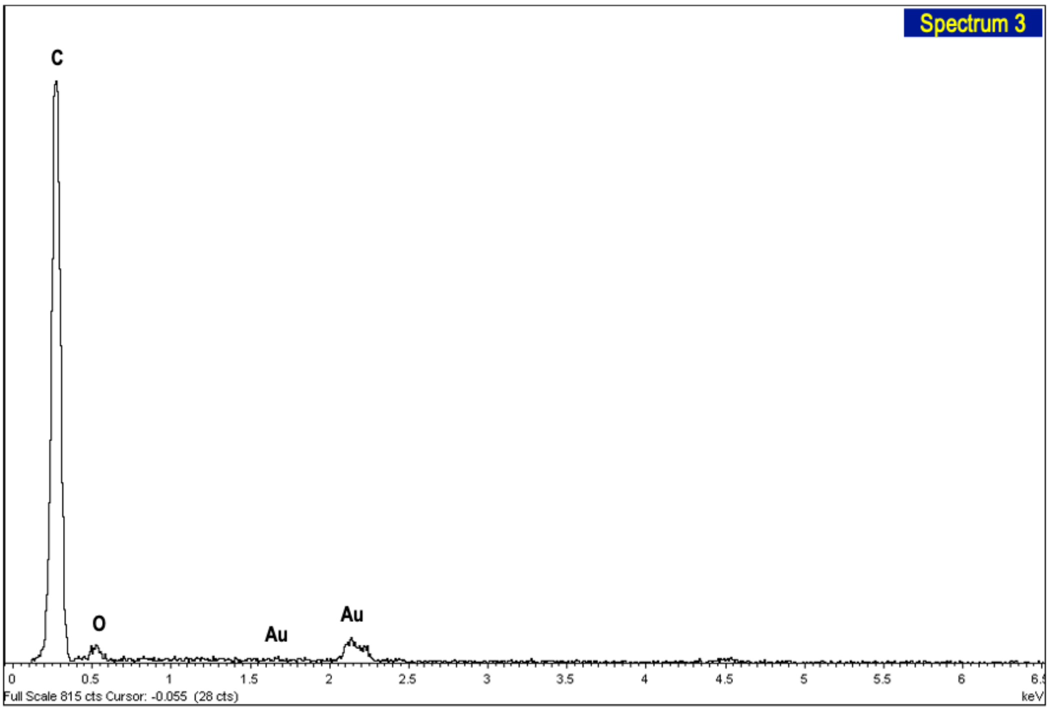

Figure S4. SEM-EDS analysis of the HDPE MPs after photocatalysis at 0 °C and pH 3 for 50 hours (continued).

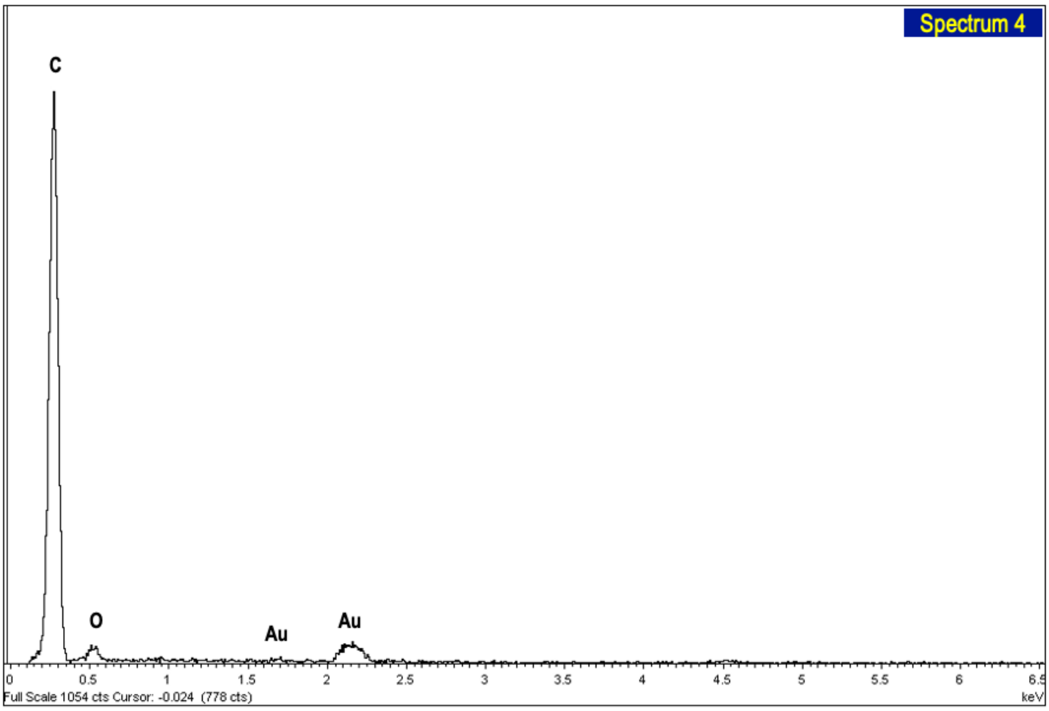

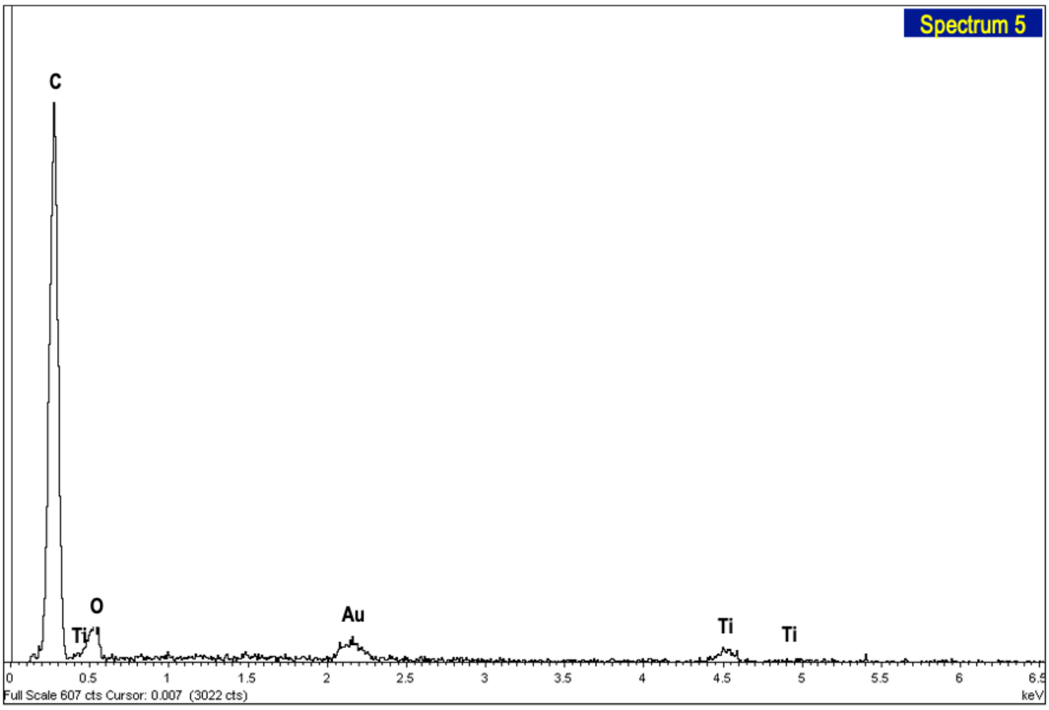

Figure S4. SEM-EDS analysis of the HDPE MPs after photocatalysis at 0 °C and pH 3 for 50 hours (continued).

Figure S5 presents a SEM micrograph at higher magnification of the HDPE MPs after photocatalysis at 0 °C and pH 3 for 50 hours. EDS microanalysis was performed to confirm the presence of semiconductor residues on the degraded plastics. Table S1 present the obtained results.

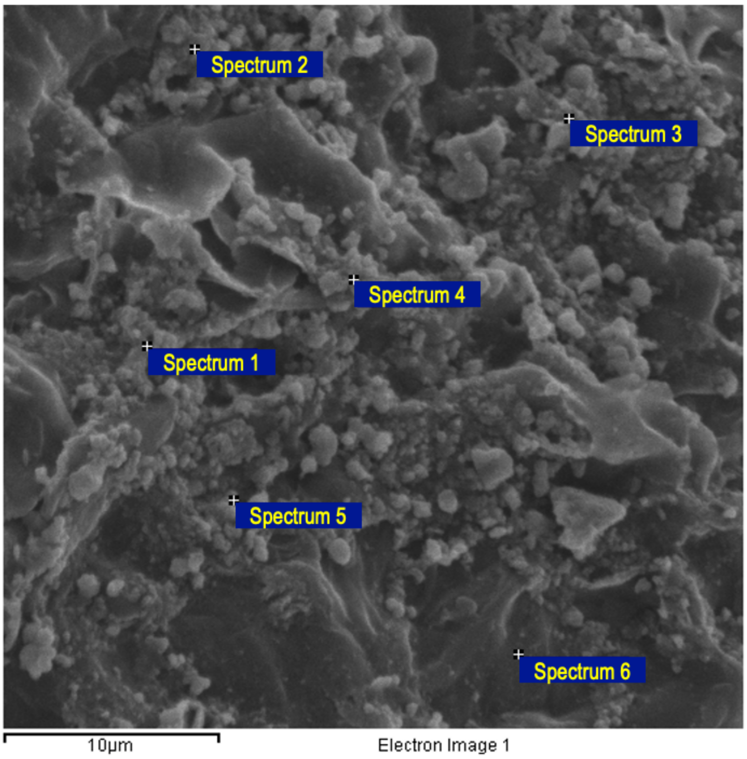

Figure S5. SEM micrograph of the HDPE MPs after photocatalysis at 0 °C and pH 3 for 50 hours (high magnification). The EDS analysis were performed in the zones indicated in the micrograph.

Table S1. EDS chemical microanalysis of the HDPE MPs after photocatalysis at 0 °C and pH 3 for 50 hours.

**EDS spectrum**	**at. %**			N_O/N_{Ti}*
	C	O	Ti	
**1**	34.47	48.69	16.84	2.89
**2**	41.85	41.9	16.25	2.57
**3**	38.62	47.52	13.86	3.42
**4**	75.57	19.92	4.51	4.41
**5**	62.61	29.33	8.07	3.63
**6**	97.93	2.16	0	-

* N_O = Number of atoms of oxygen. N_{Ti} = Number of atoms of titanium. For TiO_2, N_O/N_{Ti} = 2.

Figure S6 presents the FTIR spectra for CI calculation.

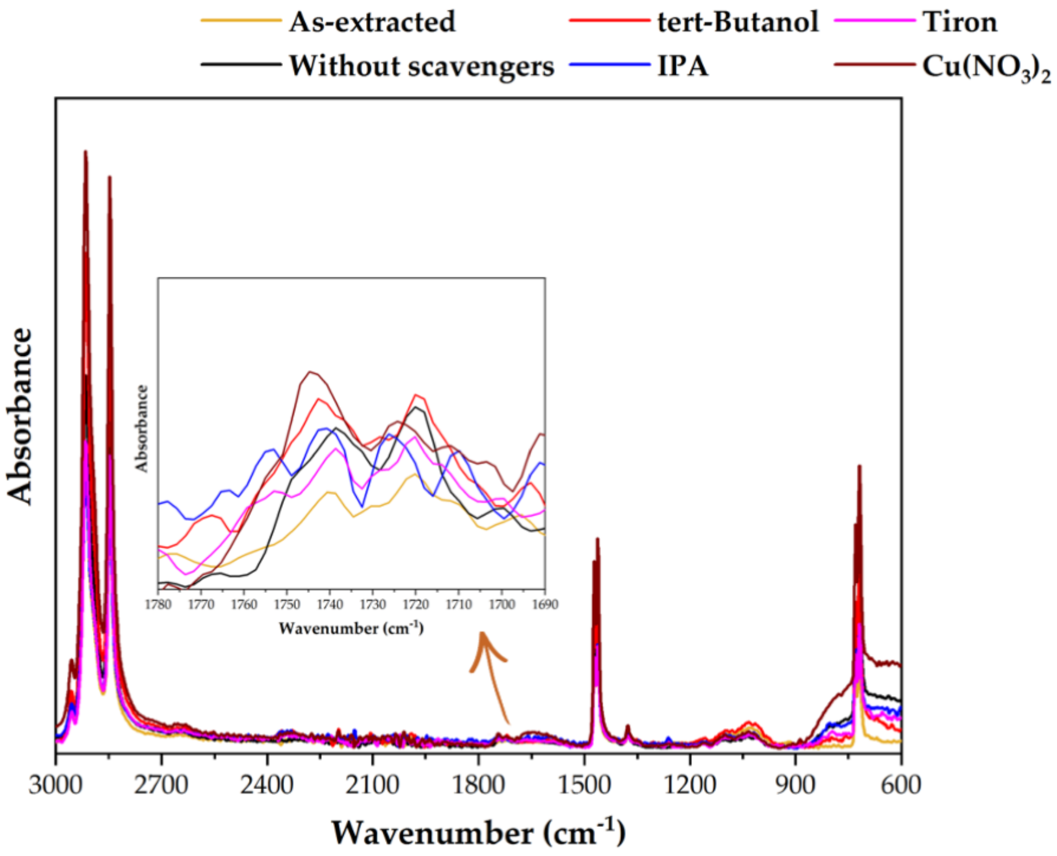

Figure S6. FTIR spectra of the HDPE MPs before and after 50 h of photocatalytic degradation with and without scavengers. Inset: carbonyl band at 1720 cm^{-1} for CI calculation.
